# Intramuscular Cavernous Hemangioma of Medial Rectus Muscle in Paediatric Age Group

**DOI:** 10.1155/2017/1076404

**Published:** 2017-03-13

**Authors:** Anuj Mehta, Shalini Butola, Mayuresh Naik, Sangeeta Abrol, Anju Kumari

**Affiliations:** Department of Ophthalmology, VMMC & Safdarjung Hospital, Ring Road, Ansari Nagar, New Delhi 110029, India

## Abstract

An 11-year-old male child presented with a mass on the nasal aspect of the right eye that has been there for the last 2 years. Extraocular movements were decreased in the right eye on levoversion, levoelevation, and levodepression. Local examination revealed a bluish mass with irregular surface and ill-defined margins located in the medial rectus muscle. The mass was 10 × 20 mm in size, firm, nodular, nontender, nonpulsatile, noncompressible, and nonreducible. MRI of the orbit revealed a well-defined mass of approximately 23 × 13 mm along the medial rectus (MR) muscle. It was hyperintense on T_2_W images with very minimal contrast enhancement. A provisional diagnosis of hemangioma or lymphangioma with intralesional haemorrhage was made. During surgical excision, the mass was found to be encapsulated by MR fibres. The MR fibres were separated, and the mass measuring 20 × 8 × 6.5 mm was removed and sent for histopathology. The histopathological examination revealed an intramuscular cavernous hemangioma.

## 1. Case Report

An 11-year-old male child presented to our hospital with a mass on the nasal aspect of the right eye that has been there for the last 2 years. It was associated with redness over the lesion which was relieved on topical medications. There was no associated pain, lacrimation, or photophobia. It has been associated with binocular diplopia on levoversion for the last 4 months. There was no history of a sudden increase in the size of the mass, pain, forward protrusion of the eyeball, or diminution of vision.

On systemic examination, no abnormality was detected. On ocular examination, the best corrected visual acuity was 6/6 in both eyes. His anterior segment and fundus examination was normal. Extraocular movements were decreased in the right eye on levoversion, levoelevation, and levodepression. Exophthalmometry using Hertel's exophthalmometer with interlateral canthal distance of 100 mm read 15 mm in both eyes.

**Figure 1:**
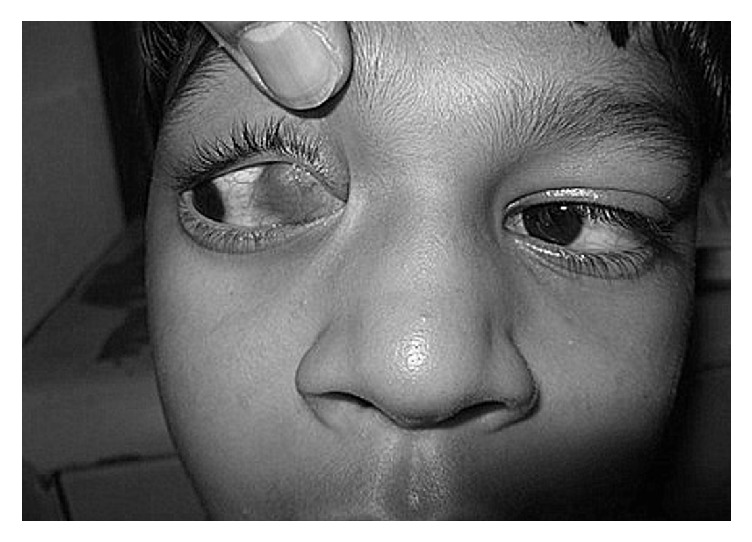
Preoperative status showing prominence of the hemangioma on dextroversion.

Local examination revealed a bluish mass with irregular surface and ill-defined margins located in the medial rectus muscle. The mass became more prominent on dextroversion. The mass was 10 × 20 mm in size, firm, nodular, nontender, nonpulsatile, noncompressible, and nonreducible (Figure 1).

**Figure 2:**
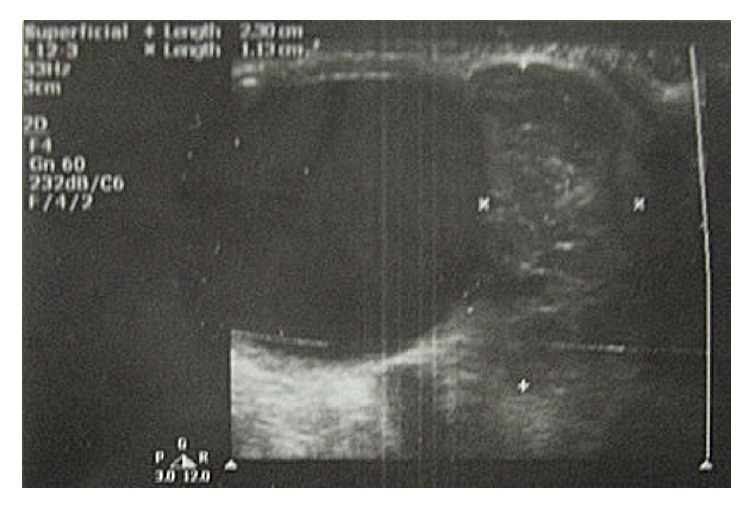
USG B-scan of orbit showing heterogenous mass in the extraconal compartment close to the insertion of the medial rectus.

**Figure 3:**
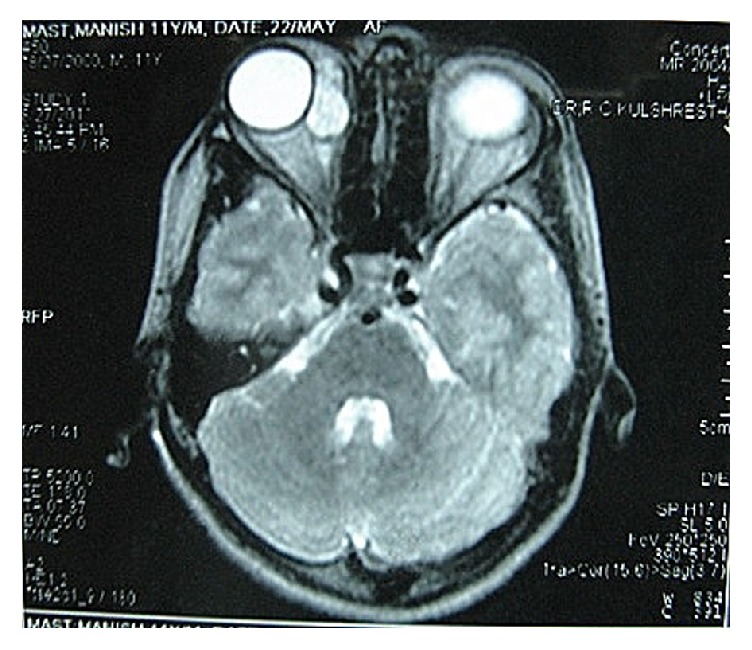
MRI scan of orbit MRI of the orbit revealed a well-defined mass of approximately 23 × 13 mm along the medial rectus muscle.

USG B-scan revealed a heterogenous mass approximately 23 × 13 mm in extraconal compartment close to the insertion of the medial rectus (MR) muscle with few hyperechoic flecks (Figure 2). MRI of the orbit revealed a well-defined mass of approximately 23 × 13 mm along the medial rectus (MR) muscle. It was isointense on T_1_W and hyperintense on T_2_W images with very minimal contrast enhancement (Figure 3). The patient initially consulted a local ophthalmic surgeon elsewhere, where it was initially diagnosed as cysticercosis and treated with albendazole and oral steroids.

A provisional diagnosis of hemangioma or lymphangioma with intralesional haemorrhage was made. As the patient had already received a course of oral steroids without any response and there was no evidence of active flow in the lesion, a decision to carry out excision biopsy was taken.

**Figure 4:**
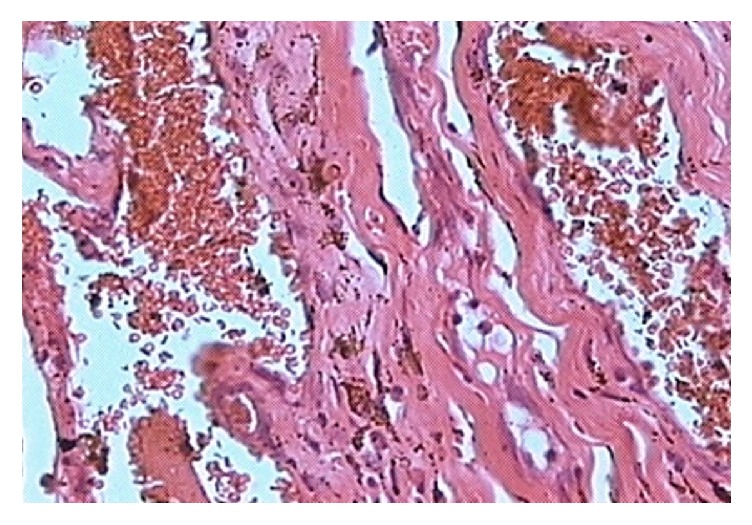
Histopathological section showing cavernous hemangioma.

During surgical excision, the mass was found to be encapsulated by MR fibres. The MR fibres were separated and the mass was removed completely. The MR fibres were sutured together with 6-0 vicryl suture. The mass measuring 20 × 8 × 6.5 mm was removed and sent for histopathology. The histopathological examination revealed an intramuscular cavernous hemangioma (Figure 4). The patient was started on oral steroids for two weeks to ameliorate the postoperative edema and fibrosis.

## 2. Discussion

Intramuscular hemangiomas (IMHs) are nonmetastasizing, benign, hamartomatous, congenital neoplasms which remain unremarkable for long but may start growing in the second and third decade of life [1, 2]. Allen and Enzinger classified them into three types based on histopathological features into capillary (50%), cavernous (29%), and mixed (21%). IMHs are rare tumors [3]. Less than 1% of hemangiomas occur in skeletal muscles and more than 20% occur in the head and neck region. The IMHs in extraocular muscles are very rare [1–3]. The aetiology of these lesions is unknown although trauma or hormonal changes are considered important factors in the proliferation of embryonic vascular tissue. Even a congenital theory has been proposed on account of the high incidence of hemangiomas during the first years of life. Only six such cases are recorded in the literature (Table 1).

Christensen et al. reported a case of a 21-year-old female with a 10-year history of proptosis. She was operated on thrice in 9 yrs without reaching a diagnosis. Enucleation of the eye ball with excision of the affected muscle was done which revealed mixed IMH involving four of the six muscles [4].

Kiratli et al. reported two cases of isolated IMH of extraocular muscles: one in a 3-year-old child with involvement of lateral rectus muscle and the other in a 40-year-old man with involvement of the medial rectus muscle. Both of them presented with proptosis and lid swelling. On histopathological examination, the child was diagnosed to have capillary type IMH and the adult man was diagnosed to have mixed type IMH [5].

In our case, the medial rectus was involved. The mass could be excised completely and, on histopathological examination, a purely cavernous type of IMH was noted. In six cases reported till date, 2 cases had mixed type, 2 had capillary type, and 2 had cavernous type (in an adult 63-year-old patient and a 31-year-old pregnant female) on histopathological examination [4–7]. Our case was unique because it is the first case of a purely cavernous hemangioma in a child.

The armamentarium of management options narrows down to just two: medically by systemic steroids and surgically by complete excision. Systemic steroids can reduce the bulk of the tumor but IMHs of extraocular muscles are insensitive to systemic corticosteroids because of encapsulation and presence of cavernous elements which are resistant to corticosteroids [3]. Open surgical excision, including an adequate rim of surrounding healthy tissue, is the treatment of choice for management of such lesions, although recurrences ranging from 9 to 28% have been described even after wide resection of a cuff of normal muscle around the tumor [8]. In our case, no recurrence was noticed even after 3 years of regular follow-up.

## Figures and Tables

**Table 1:** Review of cases of “intramuscular hemangiomas” in extraocular muscles.

Year	Author	Age	Extraocular muscle involved	Histopathologic type of IMH
2002	Christensen	21 yrs	MR, LR, IR, SO	Mixed
2003	Kiratli	3 yrs	LR	Capillary
2003	Kiratli	40 yrs	MR	Mixed
2006	Kim	63 yrs	SR	Cavernous
2009	Lee	31 yrs	MR	Cavernous
2014	Charles	25 yrs	IO	Capillary

MR: medial rectus; LR: lateral rectus; IR: inferior rectus; SR: superior rectus; IO: inferior oblique; SO: superior oblique.
